# Understanding Mechanical Properties of *Nothofagus alpina* (Poepp. & Endl.) Oerst. Wood Through Controlled Freeze–Heat Treatments: Linking Physical, Chemical, and Structural Changes

**DOI:** 10.3390/ma19061275

**Published:** 2026-03-23

**Authors:** Rodrigo Valle, Romina E. Inostroza, Luis Soto-Cerda, Wilmer Bueno-Silva, Marcelo Muñoz-Vera, Víctor Tuninetti, Ricardo I. Castro

**Affiliations:** 1 Construction Multidisciplinary Research Group, Facultad de Arquitectura, Construcción y Medio Ambiente, Universidad Autónoma de Chile, Talca 3460000, Chile; rodrigo.valle@uautonoma.cl; 2 Facultad de Ciencias de la Ingeniería, Campus San Miguel, Universidad Católica del Maule, Avenida San Miguel 3605, Talca 3460000, Chile; rominainostroza12@gmail.com; 3 Construcción y Medio Ambiente, Construction Multidisciplinary Research Group, Facultad de Arquitectura, Universidad Autónoma de Chile, Talca 3460000, Chile; luis.soto@uautonoma.cl; 4 Departamento de Ciencias Ambientales y Recursos Naturales, Universidad de Alicante, San Vicente del Raspeig 03001, Spain; 5 Departamento de Obras Civiles, Facultad de Ciencias de la Ingeniería, Campus San Miguel, Universidad Católica del Maule, Avenida San Miguel 3605, Talca 3460000, Chile; wbueno@ucm.cl; 6 Facultad de ingeniería, Universidad Santo Tomas, Sede Talca 3460000, Chile; marcelomunoz132@gmail.com; 7 Department of Mechanical Engineering, Universidad de La Frontera, Temuco 4811230, Chile; victor.tuninetti@ufrontera.cl; 8 Multidisciplinary Agroindustry Research Laboratory, Carrera de Ingeniería en Construcción, Instituto de Ciencias Aplicadas, Universidad Autónoma de Chile, Talca 3460000, Chile

**Keywords:** *Nothofagus alpina*, freeze–heat treatments, mechanical properties, chemical changes

## Abstract

Wood is a versatile material; however, it is susceptible to changes when exposed to extreme temperatures. This study investigated the physical, chemical, and mechanical properties of raulí (*Nothofagus alpina)* under different thermal stress conditions. The results showed that the moisture content at temperatures below 5 °C exhibited a significant reduction from 9.7% to 7.5% within the first 20 days. Conversely, under extreme cold (−20 °C), significant changes only occurred after 60 days, with an increase from 9.7% to 11%. At higher temperatures (50 °C, 95 °C, and 120 °C), moisture content dropped sharply after 40 days, nearing 0%. Additionally, analysis showed minor color changes in samples at low temperatures: RW2 (20 d; 5 °C, ΔE* = 3.46) and RW7 (40 d; 5 °C, ΔE* = 0.61); however, color changes were observed at higher temperatures (95–120 °C). RW15 (60 d; 120 °C, ΔE* = 37.16), indicating the degradation of cell wall polymers. Mechanical testing using three-point bending demonstrated that controlled heat treatments can improve the modulus of elasticity (MOE), modulus of rupture (MOR), and fracture energy. The most significant improvements were obtained at 120 °C for 60 days, with increases in MOE, MOR, and fracture energy of 22%, 60%, and 118%, respectively, compared to untreated wood.

## 1. Introduction

Wood is a naturally occurring material with remarkable adaptability, making it essential for engineering and building material production [1,2]. However, the intrinsic limitations of wood, such as its dimensional instability, sensitivity to environmental stressors, and susceptibility to biological deterioration, have prompted its study.

The structural integrity and chemical composition of wood depend on its polymer composition and are closely linked to its mechanical and thermal properties [3]. When exposed to heat stress, the structural complexity of wood—which is defined by the hierarchical arrangement of cellulose, hemicellulose, and lignin—undergoes significant changes. This complex 3D structure, primarily composed of cellulose (aggregated into elementary fibrils, microfibrils, and macrofibrils), hemicellulose (with principal groups like xylans, mannans, xyloglucans, and galactans), and lignin (comprising *p*-hydroxyphenyl, guaiacyl, and syringyl units [4]), can undergo progressive degradation. Temperature variations can affect its structural integrity [5,6]. These alterations are temperature-dependent, with hemicellulose starting to break down at around 160 °C and amorphous cellulose and lignin breaking down at higher temperatures [7,8]. However, prolonged exposure of wood to different cold temperatures below water’s maximum density or to temperatures near water’s boiling point could cause damage to its structure, resulting in decreased mechanical properties and changes in chemical properties [5].

With the purpose of diversifying the types of wood used in construction, there are different alternative species. *Nothofagus alpina* (Poepp. & Endl.) Oerst, also known as raulí, roblí, or ruilí, is a suitable example. It is a fast-growing arboreal species in the temperate forests of South America, in Chile and Argentina, and can grow up to 40 m in height. In Chile, the species is found in ravines and south-facing slopes of mountain and coastal ranges, at altitudes between 100 and 1200 m above sea level [9]. The trunk can reach 2 m in diameter and is largely free of branches. This wood is highly valued for the quality of its hardwood [10], and this species belongs to the same genus as other native species highly valued for the quality of their wood, including roble (*N. obliqua*), hualo (*N. glauca*), and lenga (*N. pumilio*) [11].

Formerly, Nothofagaceae species were included in Fagaceae. In that family, there are important forest species, such as *Fagus grandifolia* Ehrh. and *F. sylvatica* L. *Fagus grandifolia,* the “American beech”, which is a species from eastern United States and northeastern Mexico, and it can grow between 18 and 24 m. It is a deciduous species and its fruit is a nut, with two or three in a single bur. Its wood is frequently used for flooring, furniture, containers, railroad ties, baskets, pulp, charcoal, and rough lumber [12]. *Fagus sylvatica*, the “European beech”, is a species from central and western Europe, and it can grow between 30 and 35 m. It is a deciduous species, and its fruit is a nut, frequently with two in a single bur (it is not common to find one or three nuts per bur). Its roots are very developed, but not too deep. Its wood is strong, tough, hard, of high compressive strength and is used for furniture, flooring, paneling, veneers, railway ties, firewood, and woodenware [13]. Average density in *Fagus grandifolia* wood is 721 kg/m^3^, in *F. sylvatica* wood is 752.5 kg/m^3^ [14], and in *Nothofagus alpina* is around 550 kg/m^3^ [15].

Nevertheless, scientific knowledge of freeze–heat cycles is still lacking. Given the increased interest in employing wood products in areas with drastic temperature changes, this information gap is especially important, although it is known that raulí (*N. alpina*) does not present significant difficulties in kiln drying, with recommended temperatures under 60–65 °C, but its behavior under more extreme conditions is unknown.

From a mechanical perspective, wood has been extensively studied due to its mechanical properties, which allow its wide use in structural engineering applications [16]. The mechanical strength, stiffness, and fracture toughness of wood are determined by its chemical composition and cellular architecture, ranging from nanoscopic structures such as microfibrils to macroscopic configurations such as growth rings and xylem cells [17,18]. Among the parameters traditionally evaluated to quantify the structural behavior of wood are the modulus of elasticity (MOE), modulus of rupture (MOR), and fracture toughness, which characterize its ability to resist bending and absorb energy without catastrophic failure [19,20].

Numerous investigations are reported in the literature, where it is studied how the mechanical properties of wood are influenced by different factors such as humidity [21], aging [22,23,24], fiber orientation [25], and particularly, thermal treatments [26,27,28,29]. These treatments, applied in both cryogenic and high temperature ranges, can induce important modifications in the microstructure of wood, affecting the integrity of its constituent polymers such as hemicellulose, cellulose, and lignin [30]. This is reflected in an alteration in their mechanical properties [31]. For example, it has been reported that exposure to high temperatures causes the progressive thermal degradation of hemicellulose, which results in a loss of toughness and increased brittleness [32]. Ref. [33] investigates the fracture toughness of *Pinus radiata* D. Don subjected to heat treatment using the Double Cantilever Beam (DCB) test. Experimental procedures were performed on specimens subjected to varying temperature conditions, from 25 to 150 °C. The results reveal that the increase in heat treatment significantly influences the mechanical performance of the material. Strength and stiffness showed an increasing trend with temperature. Meanwhile, the fracture toughness showed a substantial reduction with increasing temperature. These changes in mechanical characteristics, such as decreased elasticity and increased brittleness, are due to this thermal deterioration. However, exposure to freezing temperatures causes noticeable microstructural changes at the opposite end of the thermal spectrum [34,35]. Although often leading to brittleness, the formation of ice crystals within the wood matrix and consequent cell wall shrinkage could increase stiffness [36]. These effects of freezing temperatures on wood have not yet been explored in the literature.

Therefore, despite the large increase in research on the mechanical behavior of native wood species such as spruce and pine, native South American species such as *N. alpina* have been poorly characterized in terms of their mechanical response to prolonged thermal treatments. This knowledge gap is particularly important considering the growing interest in the use of native woods for structural applications in contexts with large thermal variations, as occurs in several areas of Chile and Argentina. Currently, there are no systematic studies that analyze how the MOE, MOR, or tenacity of species such as raulí evolve when exposed to extreme static thermal conditioning (from −20 °C to 120 °C) for prolonged periods of time. In this context, the present research seeks to fill this gap by means of a multi-scale analysis linking controlled thermal treatments with the mechanical performance of raulí. Through bending tests on samples exposed to different levels of temperature and time, it is intended to understand how their internal structural properties are modified, laying the groundwork for future structural applications of this species in thermally demanding conditions.

Through a comprehensive analysis of raulí wood exposed to regulated static thermal conditioning, our work aims to fill this important research gap by linking these variations with mechanical performance indicators. To determine how static thermal conditioning impacts load-bearing capacity, the study simultaneously assesses important strength characteristics such as bending strength (MOR), modulus of elasticity (MOE), and compression strength parallel to the grain.

## 2. Materials and Methods

### 2.1. Wood Samples

We chose specimens without visible flaws including knots, fractures, pith, and other irregularities to design the test on *N. alpina* wood. The material is from the Araucanía Region (IX Region) of Chile (38°S–39°S). This region is known for its temperate oceanic climate, which produces slow-growing trees with high structural density with mean annual temperatures of 10–12 °C and an annual precipitation of more than 2500 mm. Harvested from secondary native forests where the trees were between 35 and 40 years old, its main composition is heartwood (about 85–90% of volume). As a result, peripheral sapwood is mostly removed to prevent biological degradation, due to Chilean sawmilling standards that require debarking before processing. Specimens were not taken from a single tree, introducing inherent biological variation in heartwood extractive content and microfibril angle that can affect mechanical responses to thermal treatment. All the samples were obtained from the same general provenance and age class to minimize geographical variability.

**Figure 1:**
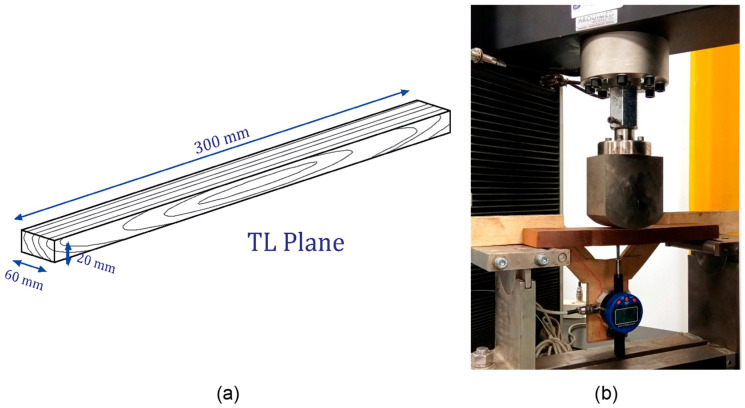
Dimensions of experimental specimens (**a**) for quasi-static bending experiments in the TL plane (**b**).

Furthermore, the materials were checked for the presence of fungus and other biological agents that can interfere with the testing process. To attain a Moisture Content (MC) of 10%, all specimens, which were 300 × 60 × 20 mm (L × T × R), were pre-conditioned at 20 ± 2 °C and 65% relative humidity [37,38]. Accurate test results require this conditioning procedure. For the quasi-static bending tests, experimental specimens of the dimensions shown in Figure 1a were manufactured to receive the load on the Transverse Longitudinal Plane (TL). For the execution of the tests, D 143-94 ASTM (American Society for Testing and Materials) standards [39] were used as reference. The tests were performed with a Laryee NE-34100 universal testing machine (Laryee Technology Co., Ltd, Xicheng District, Beijing, China). with a 100 kN load cell, which automatically recorded the applied force, while the vertical displacement was recorded by a deflectometer, as shown in Figure 1b. All mechanical tests were performed while maintaining a constant displacement rate of 1 [mm/min] and maintaining a distance between supports of 250 [mm].

### 2.2. Mechanical Properties

The MOE (modulus of elasticity) and MOR (modulus of rupture) in static bending were determined using Equations (1) and (2), respectively:

(1) $$MOE=\frac{P_{lp}\;L_{s}\;}{4\;\delta_{lp}\;b\;h^{3}}$$

(2) $$MOR=\frac{3\;Q\;{L_{s}}^{3}\;}{2\;b\;h^{2}}$$

where $L_{s}$ is the distance between supports ($L_{s}=$ 250 mm). b and h are the width and height of the samples, respectively. $P_{lp}$ is the load applied at the proportionality limit. $\delta_{lp}$ is the deflection in $P_{lp}$. Finally, Q is the maximum load is applied. On the other hand, the fracture energy $G_{f}$ will be calculated using the following Equation (3):

(3) $$G_{f}=\frac{\int_{0}^{\delta_{max}}P\;d\delta \;}{b\;h}$$

### 2.3. Assay Thermal Treatment

The selected temperature is based on distinct physicochemical transitions in wood polymers. Subzero temperatures down to −20 °C and the critical point at 5 °C governs freeze–thaw processes, where intracellular volumetric expansion (by water) induces microfractures and mechanical fragmentation of the matrix. Additionally, the temperatures from 95 °C to 120 °C target thermal degradation of components. At 95 °C (just below the atmospheric boiling point), the process is dominated by the evaporation of water and the onset of hemicellulose hydrolysis. At 120 °C, thermal scission of glycosidic bonds and dehydration of hydroxyl groups occur (depolymerization and alteration of polymer components). The samples were exposed to a range of exposure periods, treatment from 0 to 60 days. We used convex air furnaces of the BOV-T70C type, which has a temperature stability of ±1 °C, to perform the heat testing. Likewise, we used a cooling system with comparable characteristics for the cold testing. All the samples with identical dimensions and humidity levels were examined in triplicate, and each was put through a variety of tests that considered two key variables: temperature and time. A vernier was used to measure the dimensions in millimeters with an accuracy of ±0.05. Each sample’s volume and starting density were calculated using this information.

### 2.4. Density and Determination of the Moisture Content (MC)

According to ISO 3131 (1975) [40,41], the density of each sample was subsequently calculated from the moisture content (MC), where a reference sample density was derived for a nominal MC of 10%, as shown in Equation (4).

*ρ*10 = *ρ* [1 − ((1 − *k*)(*U* − 10))/100] (4)

where 10 densities were calculated to 10% MC (kg/m^3^), = density at the MC at testing (kg/m^3^), U = the MC at testing (%), and k = species-dependent correction coefficient (0.8510 × 10^−3^ per ISO 3131 1975) [40,41].

The determination of the moisture content (MC) was calculated by direct measurement with a digital hygrometer, model MM-1010 wood moisture meter (TLETEK) ZQFEE, Shenzhen, China).

Regarding the wood samples, the measurements were made in both the longitudinal direction and transverse direction at room temperature. The moisture was measured six times for the longitudinal and six times for the transverse direction for each amount of MC of the wood samples. All the MC measurements were performed in the middle of the width of each tested surface.

### 2.5. Measurement of Color Parameters

The color of the wood samples was measured with a portable spectrophotometer (Nix Pro 2, Nix Sensor Ltd., Hamilton, Ontario, Canada) with a 14 mm aperture six times on two distinct faces of the sample. The color obtained was analyzed according to the CIELab color scale (space defined by the International Commission on Illumination, CIE (Commission Internationale de l’Éclairage) that gives the color in coordinates (L, a, and b), where L* is luminance from black (0) to white (100), a* from green (−) to red (+), and b* from blue (−) to yellow (+) [42].

After obtaining the measurements of the color coordinates (L, a, and b), the difference in brightness is represented as ∆L and the chromatic coordinates as ∆b and ∆a. With these data, the total color change ∆E can be obtained from each sample analyzed (see Table 1 and Equation (5)):

(5) $$∆E={\left[{\left(∆L\right)}^{2}\;+\;{\left(∆a\right)}^{2}\;+\;{\left(∆b\right)}^{2}\right]}^{1/2}$$

### 2.6. Statistical Analysis

All experiments were performed in triplicate, and results were expressed as mean ± standard deviation (SD). Statistical analysis was performed using StatGraphics Centurion XVI software. A two-way analysis of variance (ANOVA) was conducted to evaluate the effects of storage time (three levels: 20, 40, and 60 days) and temperature (five levels: −20, 5, 50, 95, and 120 °C) as categorical factors on the response variables. The ANOVA model included both main effects (time and temperature) and their interaction term (time × temperature). With three levels for time, five levels for temperature, and three complete replicates, the degrees of freedom were allocated as follows: time (df = 2), temperature (df = 4), interaction (df = 8), and error (df = 30). Fisher’s least significant difference (LSD) test was used for multiple comparisons when significant differences were detected (*p* ≤ 0.05). A confidence level of 95% (α = 0.05) was considered for all statistical tests.

Additionally, significant effects were detected in the mechanical properties evaluated as MOE, MOR, and fracture energy. Tukey’s honestly significantly different (HSD) post hoc test was applied to compare temperatures within each exposure time. Treatments with the same letter do not show significant differences.

## 3. Results and Discussion

### 3.1. Moisture Content (MC), Density Analysis, and Changes in the Wood

A study was conducted to evaluate the properties of *N. alpina* wood under various thermal stress conditions. The effects of temperature and exposure duration on the wood’s physical, chemical, and mechanical properties were analyzed. Moisture content measurements revealed a decrease below initial levels in all specimens (see Table 2). At temperatures below 5 °C, *N. alpina* wood samples exhibited a significant moisture reduction from 9.7% to 7.5% within the first 20 days. Conversely, under extreme cold (−20 °C), significant changes only occurred after 60 days, with moisture content increasing from 9.7% to 11%. This increase after extended exposure to −20 °C is probably the result of complex, thermally induced internal redistribution and phase changes in the existing water. Gradual and continuous freezing conditions can cause multiple simultaneous phenomena. Unfrozen water in the cell walls migrates and condenses as ice in the cell lumens due to cryosorption triggered by vapor pressure gradients. Additionally, pit membranes and cell walls develop microcracks as a result of the mechanical stress caused by the slow growth of ice crystals, and the capillary redistribution of liquid water into previously unreachable voids is made easier by these new pathway. Also, a portion of bound water may become “free water” as a result of hydrogen bonds being weakened by the extended cold. All of this water is measured as a brief increase in the total moisture content [44,45].

**Figure 2:**
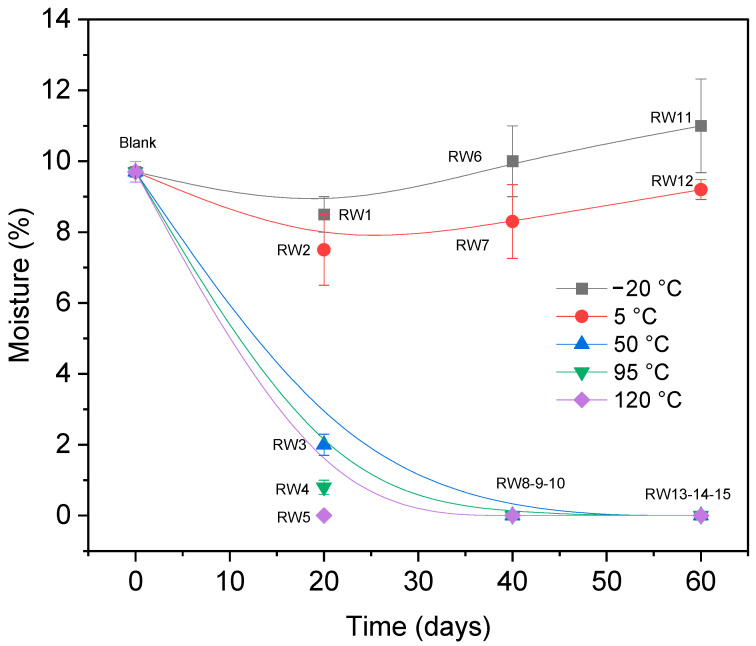
Correlation between temperature/time and moisture content (MC).

At higher temperatures (50 °C, 95 °C, and 120 °C), moisture content dropped sharply after 40 days, nearing 0% (see Figure 2), significantly altering the wood’s properties.

It is important to note that moisture content (MC) has a well-established influence on the mechanical properties of wood. Lower moisture levels generally increase stiffness and bending strength due to reduced plasticization of cell wall polymers and increased intermolecular bonding within the lignocellulosic structure. This effect has been widely documented in the literature [46]. Therefore, part of the observed increase in MOE and MOR after heat treatment can be attributed to the significant reduction in moisture content. The reported values of 0.0% correspond to moisture levels below the detection threshold of the gravimetric method and should be interpreted as extremely low residual moisture, rather than absolute zero. Therefore, the apparent mechanical improvement observed at higher temperatures and longer exposure times is likely due to a combined effect of moisture reduction and microstructural modifications, including densification and possible reorganization of the cell wall structure.

**Figure 3:**
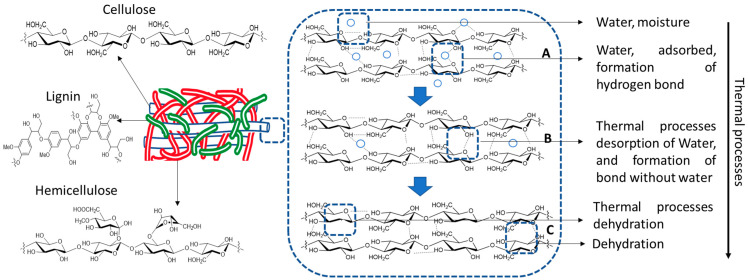
Plausible structural interaction of water and polymeric structures of wood.

Moisture loss critically affects hardwood, leading to brittleness, warping, and reduced mechanical strength. Due to the decreasing hydrogen bonds between polymeric chains, its loss weakens these interactions, diminishing dimensional stability (see Figure 3) [6,47]. Additionally, discoloration may occur due to depolymerization or structural fragmentation.

**Figure 4:**
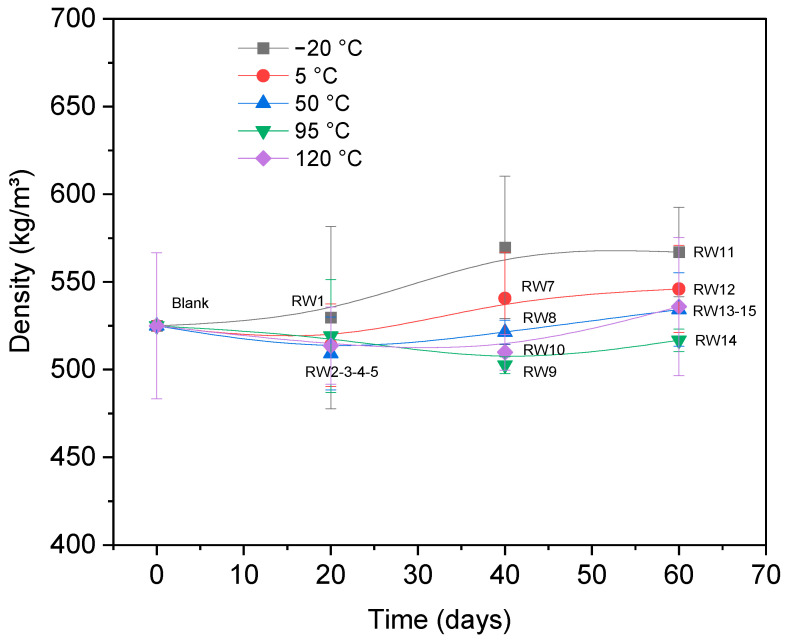
Correlation between temperature/time and density.

On the other hand, in this study, the physical changes in wood were estimated based on the density of each sample before and after thermal stress tests (see Figure 4). The initial average density was 525.9 kg/m^3^. After thermal stress, samples RW6 and RW11 (exposed to 120 °C) showed density increases of 9.29% and 9.27%, respectively. This rise is attributed to cellular collapse and dehydration-induced shrinkage, where volume reduction outweighed mass loss, compacting cell walls and reducing porosity [48].

The susceptibility of lignocellulosic components to temperature processes was analyzed. Nevertheless, the thermal protocol itself introduces inherent structural and chemical artifacts that warrant careful consideration before attributing all observed changes solely to the intended experimental variables. The volumetric expansion of intracellular and cell wall-bound water is driven by a dynamics effect that creates internal stresses that lead to irreversible microfractures and mechanical fragmentation of the wood cell wall matrix [49,50]. Regardless of the mechanisms underlying chemical degradation, this physical damage modifies the microstructure.

By removing bound water and breaking down hydrogen bonding networks between polymer hydroxyl groups, exposure to high temperatures above 95 °C, on the other hand, causes progressive dehydration, which lowers hygroscopicity and inter-component interactions [6]. As temperatures rise toward 120 °C, prolonged heat energy causes hemicellulose and amorphous cellulose to depolymerize by breaking glycosidic bonds, which is followed by lignin’s structural reorganization. Concurrently, cell wall accessibility and swelling capacity are permanently decreased by irreversible hydrogen bonding between cellulose microfibrils. Additionally, cell wall collapse brought on by high-temperature drying stresses may result in decreased dimensions (change in density) [51]. The interpretation of the main experimental variables may be complicated by these cumulative physical and chemical changes, which are inevitable changes associated with thermal exposure.

### 3.2. Color Changes Value

**Figure 5:**
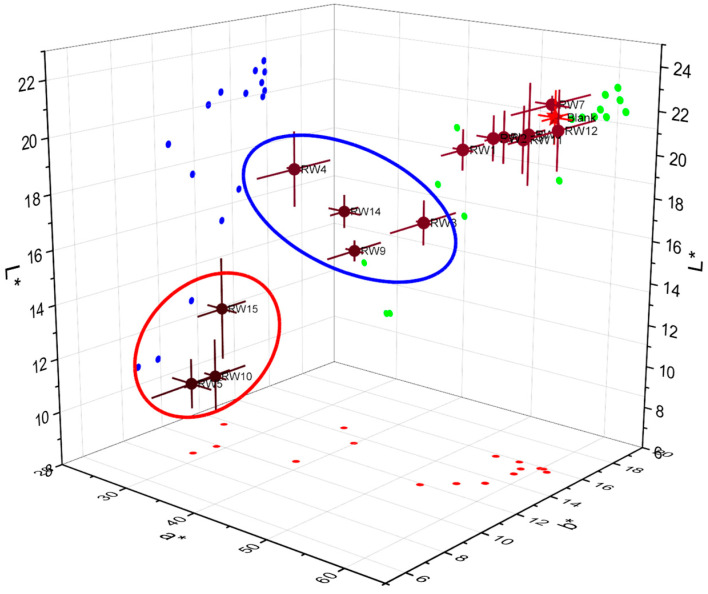
Matrix visualization of color change resulting from CIELab analysis.

The experimental results show a direct correlation of wood color changes (ΔE*) with treatment temperature and time. For samples subjected to low temperatures, the color changes were minor due to the wood components being below the threshold for heat breakdown. The samples RW2 (20 d; 5 °C, ΔE* = 3.46) and RW7 (40 d; 5 °C, ΔE* = 0.61) show that extended exposure to temperatures close to ambient causes very little color change, indicating that these circumstances maintain the wood’s original look (see Table 3 and Figure 5).

However, the color changes were observed at higher temperatures (95–120 °C), which is consistent with the results described for thermal modification thresholds [52]. The most noticeable alteration was seen in sample RW15 (60 d; 120 °C, ΔE* = 37.16), which showed a darkening (distinct color changes) linked to lignin modification and hemicellulose breakdown [53] (see Figure 5; red circle). Although the results indicate that the start begins at lower temperatures (95 °C) with longer durations, this is like the study’s finding that temperatures ≥ 160 °C induce large ΔE* rises [45]. Through cumulative thermal deterioration, the 40-day treatments at 95 °C (RW9, ΔE* = 21.67) and 120 °C (RW10, ΔE* = 34.33) additionally demonstrate that temperature and time work in concert to induce color changes.

Unpredictable ΔE* values (RW1 = 5.70, RW6 = 1.93, RW11 = 2.11) were obtained from the subzero treatments (−20 °C), suggesting that freezing-induced physical changes (like cell wall microfractures) could affect light-scattering properties without resulting in chemical changes. Color parameters stayed constant at −20 °C for all exposure times (20, 40, and 60 days), but visual appearance changed irregularly, resulting from the creation of multiple interfaces with varying refractive indices by microfractures at the cell wall level, which encourage optical scattering that modifies perceived color without changing chromophore compounds (without change in ΔE; Table 3). Freeze-induced microfractures produce heterogeneous light diffusion patterns, which account for the observed irregular color distribution, in contrast to chemical degradation, which usually results in uniform color shifts in the wood by the degradation of polymers and the formation of low-molecular-weight compounds produced during the depolymerization [54].

### 3.3. Flexural Stiffness

#### Modulus of Elasticity (MOE), Modulus of Rupture (MOR), and Energy Absorption Capacity

**Figure 6:**
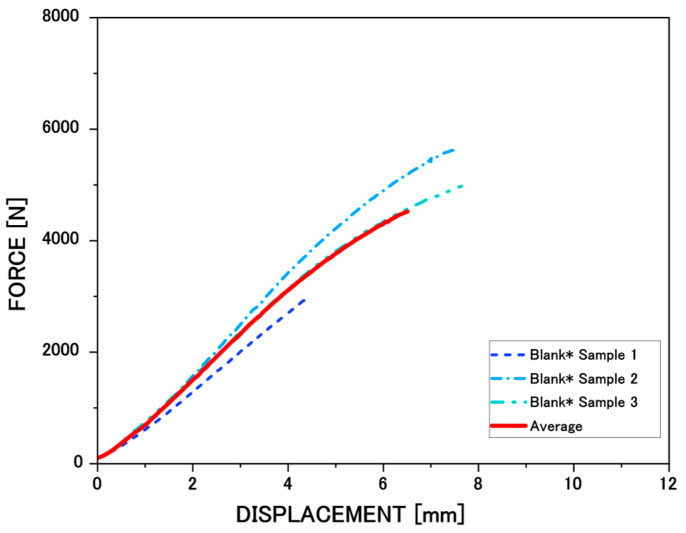
Representative force–displacement curves obtained from three independent replicate specimens without heat treatment (Blank Samples 1–3).

The mechanical behavior of *N. alpina* wood was evaluated by means of force–displacement curves obtained from three-point bending tests. These curves provide detailed information on stiffness, strength, and fracture energy for each thermal treatment condition and under different exposure times. Figure 6 shows the force–displacement curve of the Blank Sample. For the control condition (Blank* Samples), three independent samples without heat treatment were analyzed to ensure statistical reliability. Figure 5 shows the individual force–displacement curves of the three control replicates (Samples 1, 2, and 3), together with the corresponding average curve. It presents the characteristic mechanical behavior of unmodified wood, maintaining a well-defined initial elastic zone, followed by a sharp drop in load after rupture, which evidences an average MOE and MOR of 4.2 [GPa] and 50.9 [MPa], respectively, and a moderate average energy absorption capacity of 11,159.3 [J/m^2^]. This curve serves as a reference for analyzing the effect of the different thermal treatments presented in Figure 6.

**Figure 7:**
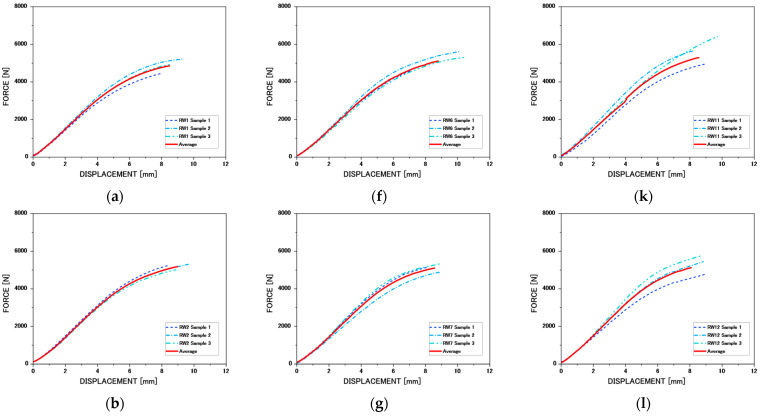

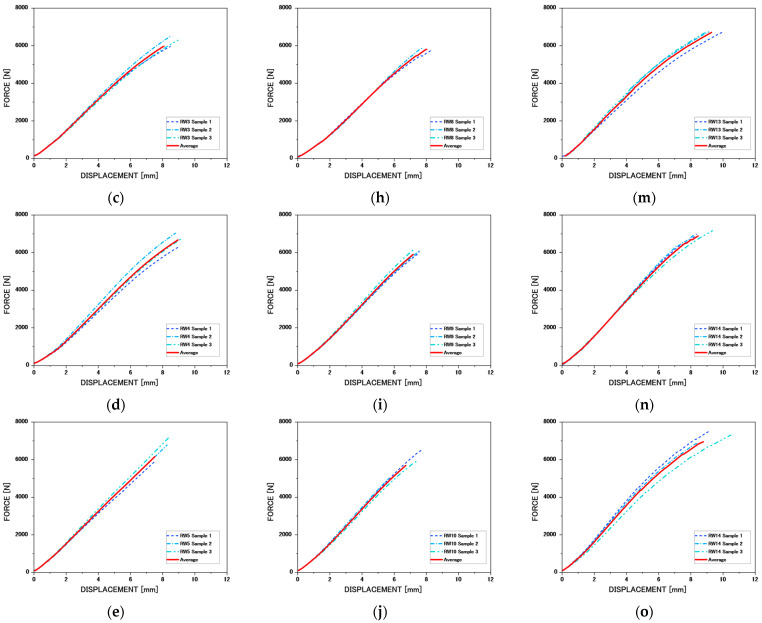
Comparative matrix of load–displacement curves obtained from bending tests on heat-treated wood samples. Each subfigure corresponds to a specific treatment temperature (−20; 5; 50; 95; 120 [°C]), while within each one the results are shown according to three exposure times (20; 40; 60 days). (**a**) RW1; (**b**) RW2; (**c**) RW3; (**d**) RW4; (**e**) RW5; (**f**) RW6; (**g**) RW7; (**h**) RW8; (**i**) RW9; (**j**) RW10; (**k**) RW11; (**l**) RW12; (**m**) RW13; (**n**) RW14; (**o**) RW15.

On the other hand, as the treatment temperature (from −20 °C to 120 °C) and exposure time (20, 40, and 60 days) increase, a progressive variation in the mechanical behavior of the samples is observed in Figure 7.

Under cryogenic exposure (−20 °C), a slight increase in initial stiffness (MOE) is observed, which may be associated with cell wall contraction and reduced molecular mobility within the lignocellulosic matrix, as previously reported in Zhao et al. [34] and Li et al. [36] for wood at low temperatures. These studies suggest that lowering the temperature limits molecular movement and may temporarily increase elastic stiffness. However, some force–displacement curves show a more pronounced drop after maximum load, indicating more abrupt crack propagation once failure has begun. It is important to distinguish between brittleness after maximum load and total fracture energy. In this study, fracture energy was calculated as the area under the force–displacement curve normalized by the fracture surface area. While crack propagation may appear more abrupt under prolonged cryogenic exposure, the higher pre-peak load levels observed in some specimens may result in equal or slightly higher total fracture energy compared to the untreated condition. Therefore, cryogenic treatment does not necessarily reduce the total energy absorption capacity, but it can modify the balance between stiffness, peak strength, and post-peak dissipation mechanisms.

In the case of samples treated at 5 °C and 50 °C, the curves show a slight decrease in MOE and peak maximum load (MOR), indicating moderate structural weakening. This behavior may be related to the partial rearrangement of the hemicellulose chains without reaching significant degradation, affecting the matrix without collapsing the structural integrity. On the other hand, at higher temperatures, especially 95 °C and 120 °C, a significant increase in stiffness and maximum load capacity is evident at all durations evaluated. Moreover, this increase is more evident as the time of each treatment increases (60 days). The curves show a higher initial slope, a higher maximum load capacity, and a steeper post-fracture drop. This behavior may result from a combined effect of hemicellulose modification and significant moisture content reduction induced by thermal exposure, which is known to increase stiffness and strength by reducing cell wall plasticization, which, according to Alén et al. [30] and Peng et al. [32], begins to decompose at temperatures close to 100 °C, generating a stiffer internal structure and weakening the structural bonds between fibers. In addition, at 120 °C, partial degradation of hemicellulose and the dehydration of wood may begin, further compromising fracture toughness. The effect of the exposure time to heat treatment is relevant as it increases from 20 to 60 days. For example, the curves corresponding to samples treated at 95 °C and 120 °C for 60 days show the greatest increase in stiffness and strength, evidence of a correlation between the duration of treatment and this mechanical improvement. This result is consistent with that reported by Gaff et al. [29], where an increase in fracture toughness was observed in heat-treated wood, which can be attributed to the loss of cohesion between microfibrils and the reduction in the available hydroxyl bonds due to the decrease in physically weak or chemically strong bound water [55], which favors the deformation capacity before fracture.

The evolution of fracture energy under heat treatment can be interpreted more accurately within the framework of fracture mechanics. In a failure dominated by bending, fracture energy represents not only the energy required for crack initiation but also the energy dissipated during its propagation within the fracture process zone (FPZ) [46]. Wood behaves as a quasi-brittle material, where microcracking, fiber detachment, interfacial slippage, and fiber bridging mechanisms contribute significantly to energy absorption beyond the maximum load. The measured fracture energy, calculated as the area under the force–displacement curve normalized by the fracture surface area, reflects the material’s resistance to crack propagation rather than just its maximum strength. In quasi-brittle materials, the FPZ plays a key role in stabilizing crack growth, and classical linear elastic fracture mechanics do not fully capture these nonlinear energy dissipation processes [46]. The development of fiber bridges across cracks increases crack tip resistance and delays unstable propagation, thereby improving post-peak energy dissipation. Under controlled thermal exposure, moderate degradation of hemicellulose, combined with partial densification of the cell wall, can increase interfibrillar friction and improve stress transfer efficiency, which could promote more stable crack propagation. These microstructural changes can facilitate crack bridging mechanisms and the extension of process zones, which explains the observed increase in fracture energy. When degradation remains limited and does not induce severe embrittlement, the increase in MOE and MOR may be accompanied by greater resistance to cracking, which explains the increase in mechanical performance parameters at elevated temperatures. However, excessive thermal degradation would likely reduce the integrity of the polymer chain and limit the bridging capacity of the fibers, resulting in brittle fracture behavior and reduced energy.

**Figure 8:**
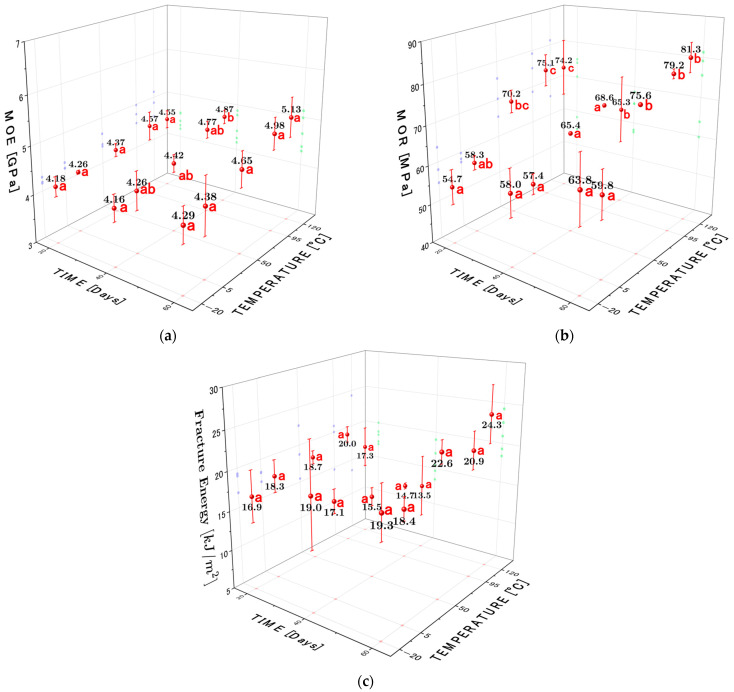
Evolution of (**a**) MOE, (**b**) MOR, and (**c**) fracture energy as a function of treatment temperature and exposure time. Error bars represent standard deviation (n=3). Different letters within the same exposure time indicate statistically significant differences among temperatures (Tukey’s HSD, p≤0.05).

On the other hand, Figure 8 and Table 4 show the evolution of three key mechanical indicators: modulus of elasticity (MOE), modulus of rupture (MOR), and fracture energy versus different heat treatment conditions and exposure times. The results show that the thermal treatments, both cold and heat, induce significant changes in the MOR, the MOR, and the energy absorbed during the fracture of raulí wood, which correlates with the alterations shown in its chemical composition. It can be observed that low temperatures generate cellular compression stiffness, which favors brittleness, evidencing a lower MOE and MOR of 4.18 [GPa] and 54.65 [MPa], respectively. However, by increasing the exposure time to 60 days at this −20 °C temperature, the MOE and MOR experience a slight increase of 4.29 [GPa] and 63.75 [MPa], respectively. Similarly, the fracture toughness is influenced, and the results reveal that the pre-fracture energy absorption undergoes a slight change from 16,902.50 to 19,271.45 [J/m^2^] from 20 to 60 days of exposure time at −20 °C temperature.

ANOVA analysis revealed that both temperature and exposure time significantly affected MOE and MOR p≤0.05, while fracture energy was mainly influenced by exposure time. The interaction term was not statistically significant for MOE or MOR. Post hoc comparisons using Tukey’s HSD test showed that significant differences were observed between temperatures, as shown in Figure 8 in the significance charts, mainly at 20 and 60 days for MOR, while differences in MOE were limited to specific exposure conditions. No statistically significant differences were detected between temperatures for fracture energy within the same exposure period.

Thus, at subzero temperatures, the MOE and MOR values remain comparable to those of the untreated reference (Blank Sample), with a slight increase with time. This increase is probably due to the shrinkage and densification of the cell wall caused by internal ice formation, which improves the bearing stiffness without seriously affecting ductility.

On the other hand, at intermediate temperatures (5 °C and 50 °C), the results show that the stiffness and strength remain stable or change slightly, which can be attributed to a slight reorganization of the polymers of wood and the water molecules without significant chemical degradation. These conditions seem to preserve the structural equilibrium of the wood without inducing abrupt failure behavior. For example, at 5 °C, the MOE shows a moderate increase (4.26 to 4.38 [GPa]) with increasing exposure time from 20 to 60 days, while the MOR increases from 58.25 to 59.79 [MPa]. The fracture energy ranges from 18,279.31 to 18,359.51 [J/m^2^]. At 50 °C, the mechanical behavior changes more pronouncedly: the MOE increases to 4.65 [GPa] and the MOR reaches 75.58 [MPa] after 60 days. This indicates that the gentle increase in temperature improves the ability to transmit stress, possibly due to the reorganization of the hemicellulose chains. The fracture energy after 60 days of treatment at 50 °C increases to more than twice that of the control (22,592.08 [J/m^2^]), demonstrating a very favorable balance between stiffness and ductility.

To explain the simultaneous increase observed in MOE, MOR, and fracture energy at high temperatures, we propose a multiscale interpretation that integrates chemical and microstructural changes. First, the selective degradation of hemicelluloses during heat treatments reduces the fraction of low-molecular-weight polymers and amorphous matrix, which increases the relative proportion of cellulose and lignin and may promote more efficient microfibril packing, increasing cell wall stiffness. This selective loss of hemicellulose has been reported and associated with changes in mechanical properties after heat treatment [46,56]. Similarly, under certain conditions (temperature/humidity control), dehydration and shrinkage of the matrix lead to local densification of the cell wall, reducing the lumen and increasing interfibrillar contact, resulting in an increase in effective density and improved stress transfer between fibers; this can increase both the elastic modulus and ultimate strength. The literature shows that these processes of controlled densification or thermal–hydromechanical compaction have shown notable improvements in stiffness and toughness in treated wood [57,58]. Additionally, previous experiments on wood subjected to loads and heat treatments show that a local decrease in the microfibril angle and fibril orientation produces an increase in stiffness [59]. This is because changes in the nanoscopic geometry of the wall or reorganization of the microfibrils increase the effective axial component of the cellulose, resulting in an increase in MOE. Meanwhile, the local decrease in the microfibril angle can also improve the transmission of stresses to the fracture energy components, contributing to an increase in MOR and fracture toughness. Thus, under the heat treatment conditions used in this study, there is a combination of the partial loss of hemicellulose (reduction in the amorphous fraction), shrinkage, the partial compaction of cell walls (densification), and local modification of/reduction in the microfibril angle. This results in a material with greater packing and better stress transfer, allowing for a simultaneous increase in stiffness, ultimate strength, and energy absorbed before failure. However, this behavior depends critically on the treatment conditions: if chemical degradation is too intense, then embrittlement will dominate, and toughness will decrease.

Finally, at the highest treatment temperatures (95 °C and 120 °C), a substantial improvement in mechanical performance is observed, especially with 60 days of exposure. On the one hand, at 95 °C, an MOE of 4.98 [GPa], an MOR of 79.16 [MPa], and an energy absorption of 20,888.89 [J/m^2^] are achieved. Then, at 120 °C, an MOE of 5.13 [GPa] (+22% compared to the Blank sample), an MOR of 81.28 [MPa] (+60% than to the Blank* sample), and a fracture energy absorption of 24,296.33 [J/m^2^] (+118% compared to the Blank* sample) are achieved. This behavior consists of the controlled degradation of hemicellulose, leading to matrix densification and improved fiber bonding, as concluded by Alén et al. [30] and Gaff et al. [29]. This improvement in the mechanical behavior of raulí wood highlights the importance of the controlled thermal degradation of hemicellulose and the partial depolymerization of lignin in increasing molecular packing and stress transfer. Contrary to the common perception that high-temperature treatments reduce toughness, these results demonstrate that, under controlled exposure, thermal exposure resulted in increased stiffness, strength, and fracture energy, likely due to a combined effect of moisture reduction and microstructural modification.

These results are particularly relevant from the perspective of wood mechanics, since it demonstrates that heat treatments, with properly controlled temperature and duration, can significantly improve both the stiffness and toughness of hardwood species such as raulí, which is still in its infancy. The ability to simultaneously increase MOE, MOR, and fracture energy is highly desirable for applications requiring resistance to dynamic loads and impacts. In addition, thermal conditioning is highlighted as a potential method for improving the mechanical performance of native woods.

### 3.4. Effect Relationships of Heat–Freeze Treatment on MOE, MOR, and Fracture Energy in Nothofagus Alpina

The analysis of the correlation between the modulus of elasticity (MOE), modulus of rupture (MOR), and fracture energy was based on small specimens. This approach aimed to quantify how the intrinsic heterogeneity of wood, derived from its irregular fiber distribution, anatomical variability, and natural density fluctuations, affects the magnitude and consistency of these mechanical properties.

**Figure 9:**
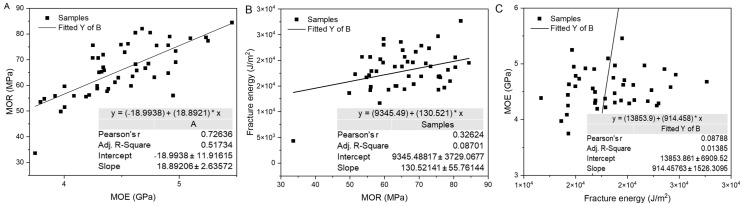
Relationship between mechanical parameters based on linear regression analysis (**A**) MOR v/s MOE, (**B**) Fracture energy v/s MOR and (**C**) MOE v/s Fracture energy.

The results of the relationship between MOE and MOR showed a strong correlation (R^2^ = 0.73) across samples treated at different temperatures and exposure times. However, this interaction featured negative y-intercepts, indicating that MOR increased more than proportionally with increases in MOE (see Figure 9A for regression results). These results align with the fundamental definitions of these properties: MOE measures the material’s stiffness (i.e., how much it deforms under a load within its elastic range), while MOR measures the maximum stress it can withstand before failure. Consequently, the correlation between them and the decrease in values is influenced by the depolymerization or fragmentation of polymeric compounds in the wood and moisture loss due to dehydration.

In contrast, Figure 9B,C shows a low correlation between fractured energy (the total energy the material can absorb before fracturing) and MOE. This low or even inverse correlation is explained by the fact that MOE is an elastic, linear property, that depends primarily on the bonds between the wood components, meaning a very rigid material (high MOE) is often brittle, as it lacks mechanisms to dissipate energy beyond its elastic limit.

Conversely, a tough material (high fracture energy) must be able to deform plastically and possess energy dissipation mechanisms, such as the formation of microcracks through depolymerization, fragmentation, or fiber degradation, which explains why the relationship between MOR and fracture energy has a higher R^2^ of 0.32 compared to the MOE–fracture energy relationship with an R^2^ of 0.087. These low correlations indicate that stiffness and ultimate flexural strength are not reliable predictors of fracture energy in our dataset. Fracture energy depends largely on post-peak dissipation mechanisms (fiber detachment and matrix fragmentation), which are governed by microstructural changes and chemical alterations of cell wall polymers induced by heat treatments, rather than by elastic stiffness alone. Similar observations have been reported in studies of thermally modified woods: while MOE/MOR may increase or remain stable, fracture energy may follow a different trajectory depending on the severity of the treatment and the species [5].

The dissociation between SEA and MOE/MOR can be understood from a mechanical perspective: thermal modification can increase stiffness and ultimate strength by promoting densification and improving interfibrillar packing (increasing MOE/MOR), while simultaneously altering energy dissipation mechanisms, such as reducing hydrogen bonds, thus modifying fracture propagation. Therefore, this behavior can increase or decrease fracture energy depending on the balance between fiber detachment and matrix fragmentation. Studies of wood reveal that hydrothermal/thermal modification can generate both decreases and increases in fracture metrics depending on the species and treatment range.

### 3.5. Factors Analysis

**Figure 10:**
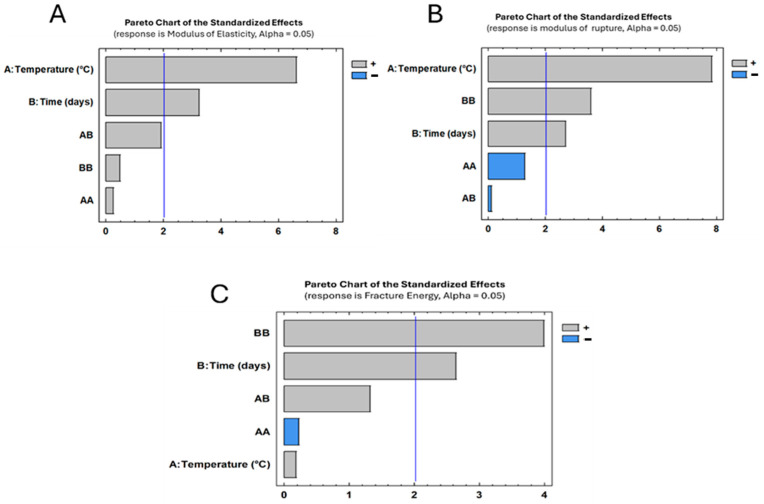
Pareto chart; factor analysis for (**A**) MOE, (**B**) MOR and (**C**) Fracture energy.

The statistical analysis of the Pareto chart confirms that the two-factor analysis of treatments on *Nothofagus alpina* showed the critical interplay between the parameters of temperature (A) and time (B) in governing mechanical properties (MOE, MOR) and energy dissipation. Both factors had significant effects. However, the temperature factor showed that higher values of this parameter may lead to decreased energy dissipation (see Figure 10, Pareto chart. Also, it can be observed that combinations of time and temperature (high temperature and prolonged time) drive a nonlinear increase in MOE due to dehydration (wood stabilization processes). Additionally, the dominance of factor B (time) increases both MOE and MOR parameters. However, the fracture energy analysis indicates that temperature does not significantly affect this parameter. Finally, the Pareto chart identifies optimal conditions where minimal energy dissipation coincides with mechanical stability, which improves at high temperatures over time.

## 4. Conclusions

This study provides a comprehensive analysis of the impact of freeze–heat treatments on the mechanical, physical, and chemical characteristics of *Nothofagus alpina* wood. The following is a summary of the main findings:

1. Variations in density and moisture content: Extreme cold (−20 °C) caused the moisture content (MC) to rise to 11% after 60 days, whereas exposure to low temperatures (5 °C) significantly reduced it from 9.7% to 7.5% in 20 days.

Rapid dehydration caused by high-temperature treatments (50–120°C) led to structural alterations and an unexpected increase in toughness, with MC approaching 0% after 40 days.

Cellular collapse and dehydration-induced shrinking caused density to rise at below-freezing temperatures, but structural densification occurred at elevated temperatures.

2. Colorimetric alterations: While high temperatures (95–120 °C) resulted in substantial darkening (ΔE* up to 37.16), indicating lignin alteration and hemicellulose breakdown, low-temperature treatments caused very slight color changes (ΔE* < 6).

Unpredictable ΔE* variations were induced by freezing conditions (−20 °C), most likely because of microfractures rather than chemical changes.

3. Improved mechanical performance: Both cryogenic and heating treatments significantly influenced the modulus of elasticity (MOE), modulus of rupture (MOR), and fracture energy. The most notable improvements were observed at high temperatures and longer exposure times:

- At 120 °C for 60 days, the MOE increased by 22%, the MOR by 60%, and the fracture energy by 118% compared to the untreated control.
- Moderate heating (50 °C, 60 days) produced a balanced improvement, with fracture energy approximately doubling while maintaining high stiffness and strength.
- Cryogenic treatment (–20 °C) produced a slight increase in fracture energy over time, indicating that the relationship between low-temperature exposure and brittleness may depend on the specific treatment conditions and wood species.

Finally, this research demonstrated that the structural integrity of rauli can be preserved under thermal exposures, even improving its mechanical properties at extreme temperatures of cold and heat (−5 and 50 °C). This allows the use of rauli as a suitable material for load-bearing applications, such as beams, pillars, and structural frameworks, where maintaining mechanical stiffness and dimensional tolerance is crucial for long-term safety and performance.

## Figures and Tables

**Table 1:** Criteria to assess the color change [43].

Parameter	Description
ΔE < 0.2	Invisibles changes
0.2 < ΔE < 2	Small changes
2 < ΔE < 3	Color changes visible by high-quality filter
3 < ΔE < 6	Color changes visible by medium-quality filter
6 < ΔE < 12	Distinct color changes
ΔE > 12	A different color

**Table 2:** Determination of density and moisture.

Sample	Time (Days)	Temp. (°C)	Density (kg/m^3^) Mean ± (SD)		Moisture (%) Mean ± (SD)	
Blank	-	-	525.9	41.6	9.7	0.29
RW1	20	−20	529.7	52.0	8.5	0.5
RW2	20	5	513.9	23.5	7.5	1
RW3	20	50	509.2	20.8	0.0	0
RW4	20	95	519.2	32.2	0.0	0
RW5	20	120	513.7	22.0	0.0	0
RW6	40	−20	569.7	40.6	10.0	1
RW7	40	5	540.7	26.4	8.3	1.04
RW8	40	50	521.3	6.9	0.0	0
RW9	40	95	502.6	4.8	0.0	0
RW10	40	120	509.9	10.2	0.0	0
RW11	60	−20	567.1	25.5	11.0	1.32
RW12	60	5	546.0	24.9	9.2	0.28
RW13	60	50	534.3	21.0	0.0	0
RW14	60	95	516.7	6.4	0.0	0
RW15	60	120	535.9	39.4	0.0	0

**Table 3:** Determination of color difference (method CIELab) (differential blank).

Sample	ΔΕ*	Change
RW1	5.98	Color changes visible by medium-quality filter
RW2	3.46	Color changes visible by medium-quality filter
RW3	9.43	Distinct color changes
RW4	22.37	A different color
RW5	35.02	A different color
RW6	1.87	Small changes
RW7	0.61	Small changes
RW8	3.25	Color changes visible by medium-quality filter
RW9	21.67	A different color
RW10	34.33	A different color
RW11	2.13	Color changes visible by high-quality filter
RW12	0.96	Small changes
RW13	6.15	Distinct color changes
RW14	24.98	A different color
RW15	37.16	A different color

**Table 4:** Mechanical properties of wood.

Sample	Time (Days)	Temp. (°C)	MOE (MPa) Mean ± (SD)		MOR (MPa) Mean ± (SD)		Fract. Energy (KJ/m^2^) Mean ± (SD)	
Blank*	-	-	4210.12	530.45	50.88	1.53	11.16	5.95
RW1	20	−20	4182.34	206.57	54.65	4.47	16.90	3.28
RW2	20	5	4264.93	40.24	58.25	1.83	18.28	2.08
RW3	20	50	4371.95	141.45	70.19	2.97	18.74	0.87
RW4	20	95	4568.96	316.98	75.15	4.23	20.03	1.07
RW5	20	120	4550.73	199.67	74.17	7.53	17.29	2.71
RW6	40	−20	4160.02	269.20	58.03	5.89	19.00	6.36
RW7	40	5	4261.17	389.02	57.40	2.52	17.12	1.47
RW8	40	50	4420.50	182.47	65.36	0.36	15.46	1.15
RW9	40	95	4774.37	177.65	68.56	0.43	14.69	0.38
RW10	40	120	4873.91	146.18	65.34	8.72	13.48	4.04
RW11	60	−20	4285.79	339.79	63.75	8.05	19.27	3.12
RW12	60	5	4380.55	557.28	59.79	5.74	18.36	1.36
RW13	60	50	4652.76	352.26	75.58	0.07	22.59	1.37
RW14	60	95	4983.09	321.98	79.16	1.14	20.89	2.33
RW15	60	120	5128.49	403.74	81.28	3.62	24.30	3.56

## Data Availability

The original contributions presented in this study are included in the article. Further inquiries can be directed to the corresponding author.
